# Characterization of highly virulent community-associated methicillin-resistant *Staphylococcus aureus* ST9-SCC*mec* XII causing bloodstream infection in China

**DOI:** 10.1080/22221751.2020.1848354

**Published:** 2020-12-01

**Authors:** Ye Jin, Xiao Yu, Yunbo Chen, Weiwei Chen, Pin Shen, Qixia Luo, Shuntian Zhang, Xiaoyang Kong, Beiwen Zheng, Yonghong Xiao

**Affiliations:** a State Key Laboratory for Diagnosis and Treatment of Infectious Diseases, National Clinical Research Center for Infectious Diseases, Collaborative Innovation Center for Diagnosis and Treatment of Infectious Diseases, First Affiliated Hospital, Zhejiang University School of Medicine, Hangzhou, China; b Department of Respiratory and Critical Care Medicine, First Hospital of Shanxi Medical University, Taiyuan, China; c Department of Laboratory Medicine, First Affiliated Hospital, College of Medicine, Zhejiang University, Hangzhou, China

**Keywords:** livestock-associated MRSA, community-associated MRSA, human-adapted, ST9-SCC*mec* XII, Whole genome sequencing

## Abstract

Previous studies have shown that livestock (LA)-MRSA ST398 evolved from a human-adapted methicillin-susceptible *S. aureus* (MSSA) clone. However, detailed information regarding ST9 is still unclear. Here, we characterized a community-associated methicillin-resistant *Staphylococcus aureus* (CA-MRSA) ST9-SCC*mec* XII isolate that has not been previously reported to cause serious disease in China. We obtained whole-genome sequences of one ST9-t899-XII isolate—ZY462471—from a patient with bloodstream infection without livestock contact. The antibiotic susceptibilities of ZY462471 were determined and the clinical information was extracted from medical notes and compared with twenty-seven previously sequenced genomes. Phylogenetic reconstruction was performed to investigate the probable host evolutionary origins of ZY462471, and the difference in resistome and virulence factors were investigated. Virulence assay was performed to evaluate the high virulence potential of ZY462471 and compare the virulence between the closest ST9 MSSA neighbours. Clinical data suggested that ZY462471 is a CA-MRSA. Phylogenetic analysis showed a much closer relationship of ZY462471 with human-associated MSSA ST9 isolates than other LA-MRSA ST9 isolates, suggesting that ZY462471 probably evolved from ST9 MSSA predecessors by acquiring an SCC*mec* cassette. Importantly, virulence assays indicated that ZY462471 was highly virulent and compared with the MSSA ST9 predecessors, ZY462471 did not show attenuated virulence. Finally, we found that ZY462471 harboured an immune evasion cluster (IEC)-carrying βC-Φ, which is typically found in human clinical *S. aureus* rather than LA-MRSA isolates, suggesting that ZY4762471 obtained the IEC-carrying βC-Φs from human clinical *S. aureus* strains. Considering its high virulence potential, this strain should be monitored to prevent more widespread dissemination.

## Introduction

*Staphylococcus aureus* is an opportunistic pathogen of humans and animals that can cause a range of infection diseases from mild skin abscesses to life-threatening conditions such as osteomyelitis, endocarditis, necrotizing pneumonia, or even septic shock [1]. Furthermore, it has now also emerged as a major cause of community-associated infections. Community-acquired methicillin-resistant *S. aureus* (CA-MRSA) infections have been rising in frequency since they were ﬁrst described in 1980s [2,3]. Apart from human infections, MRSA has also been known in animals for a long time. The first livestock-associated (LA)-MRSA was isolated in 1972 from a Belgian cow [4]. The colonization and infection of LA-MRSA have been reported in different animals, from pig farm to pasture husbandry to free-living wild terrestrial species or captive animals [5–8]. For decades, livestock-associated (LA)-MRSA has emerged in human-associated infectious diseases in patients with or without livestock contact [9]. ST398 is a major predominant lineage of LA-MRSA in many European countries [10]. Previous studies have shown that human infection and colonization rate are positively correlated with the frequency of animal contact [11,12]. However, LA-MRSA can also be transferred from livestock to humans without contact.

Unlike the predominance of the ST398 LA-MRSA in Europe and North America, ST9 is the most common LA-MRSA clone in Asia. A recent study from China collected 2420 nasal swabs from pig farms and slaughter houses in Shanghai city and the provinces of Ningxia, Henan, and Shandong and isolated 11% MRSA ST9 clones [13]. However, MRSA ST9 is rarely isolated in human infections. In China, there is still very limited information on the occurrence of infection disease by ST9 isolates in humans. Although several infections caused by ST9, both with and without livestock contact, have been reported in China [14,15], the origins of these ST9 isolates found in human subjects are still unclear, and lack detailed phenotypic analysis and further investigations. A genomic study of LA-MRSA ST398 shows that LA-MRSA ST398 had evolved from a human-adapted methicillin-susceptible *S. aureus* (MSSA) clone, and showed uptake of an SCC*mec* cassette [16]. The characteristics of ancestral human-adapted isolates were shown to be tetracycline sensitive and the human-specific immune evasion gene cluster (IEC) positive [16]. On the contrary, the LA-MRSA ST398 was shown to be tetracycline resistant and IEC negative [17]. However, detailed information regarding ST9 is still limited.

In recent years, several diseases caused by CA-MRSA ST398 have been reported, from slight localized infections to severe systemic diseases. In Japan and Denmark, cases of septicaemia caused by MRSA ST398 were also reported [18,19]. However, to our knowledge, there are no reports yet of bloodstream infections caused by MRSA ST9 in the community.

In this study, we described a severe case of bloodstream infection caused by a CA-MRSA ST9 isolate that harboured SCC*mec* XII at a tertiary hospital in the Zhejiang province of China. We sequenced the whole genome of this strain and compared it with the other ST9 isolates. In the present study, we aimed to investigate the origin and further information of this strain, based on its clinical records, genetic features, and its virulence potential. To our knowledge, the genetic feature and virulence potential of CA-MRSA ST9 strains isolated from patients with bloodstream infections has not been investigated to date.

## Materials and methods

### Collection of bacterial isolates

ZY462471 (accession number from NCBI: PRJNA669978) was isolated from the blood of a 65-year-old, previously healthy, man from Zhejiang province who presented with bloodstream infection in November 2017. He reported that 3 days before hospital admission, his right leg developed a skin abscess. He confirmed not coming into contact with livestock. The diameter of skin lesion was about 3.5 cm, and inflammatory response in the skin showed increased skin temperature, pain, and tenderness. Palpation was suggestive of skin and soft tissue infections (SSTI). Subsequently, two samples of peripheral blood were obtained for further microbiological analysis. Three days after hospitalization, the patient started showing signs of septic shock. MRSA was found in the blood culture. After a process of separation, the isolate was stored in glycerine broth.

### Antimicrobial susceptibility testing

Antimicrobial susceptibility testing of 16 antimicrobial agents including cefoxitin, ampicillin, oxacillin, gentamycin, ciprofloxacin, levofloxacin, moxifloxacin, clindamycin, erythromycin, vancomycin, linezolid, tigecycline, tetracycline, rifampicin, and sulfamethoxazole and trimethoprim (SXT) was determined in accordance with the protocols recommended by the Clinical and Laboratory Standards Institute [CLSI] [20]. *S. aureus* ATCC 29213 and ATCC 25923 strains were used for quality control per the CLSI breakpoints.

### Whole genome sequencing

Whole genome sequencing (WGS) was performed by Novogene Co., Ltd., Beijing, China. The raw data were analysed as previously described by Yu et al. [21]. *Staphylococcal* cassette chromosome *mec* (SCC*mec*) typing was performed using the web-based SCCmecFinder and ResFinder (https://cge.cbs.dtu.dk/services/SCCmecFinder/). Spa typing was performed using the wed-based SpaFinder (https://cge.cbs.dtu.dk/services/spatyper/).

### Phylogenetic analysis

In order to characterize the genetic background of ZY462471 in China, WGS was performed. The collection of ST9 isolates in China was supplemented with reads from 27 ST9 representative isolates from NCBI. Genomes of 27 ST9 isolates were downloaded from the National Center for Biotechnology Information (NCBI) for comparison of genetic features of ZY462471 (Table 1). The phylogenies of the ST9 strains were performed by ROARY software based on the core genome SNPs. Furthermore, the phylogenetic tree was constructed using MEGAX software, and the output graphic file was generated by interactive tree of life (iTOL) (https://itol.embl.de/).

**Table 1.** The detailed information of genomes of 27 ST9 isolates downloaded from the National Center for Biotechnology Information (NCBI) for comparison of genetic features of ZY462471.

Strain	Assembly	Year	Location	Host	Spa type	SCC*mec*
TSAR01	GCF_002247015.1	1998	Taiwan	Human	Unknow	V
TSAR02	GCF_002247245.1	2002	Taiwan	Human	t899	XII
TSAR03	GCF_002247005.1	2002	Taiwan	Human	t899	XII
TSAR04	GCF_002247045.1	2004	Taiwan	Human	t899	XII
TSAR07	GCF_002247125.1	2006	Taiwan	Human	t4132	XII
TSAR05	GCF_002247315.1	2006	Taiwan	Human	t800	Unknow
TSAR08	GCF_002247105.1	2010	Taiwan	Human	t899	XII
LYJ002	GCF_003693275.1	2010	Guangdong	Human	t899	XII
LYJ003	GCF_003697285.1	2010	Guangdong	Human	t899	XII
QR502	GCF_002247065.1	2012	Taiwan	Human	t899	XII
GD-G38	GCF_003665125.1	2013	Guangdong	Pig	t899	XII
GD-G33	GCF_003665115.1	2013	Guangdong	Pig	t899	MSSA
BA01611	GCF_001298325.2	2014	Shanxi	Bovine	t899	XII
NX-T55	GCF_003432345.1	2014	Ningxia	Pig	t899	Unknow
M3	GCF_003301075.1	2015	Xiamen	Pig	t899	XII
XM201503-M6	GCF_003301115.1	2015	Xiamen	Pig	t4358	XII
AL048	GCF_003310805.1	2015	Shandong	Human	t899	XII
AH027	GCF_003310835.1	2015	Shandong	Human	t899	XII
AH022	GCF_003310865.1	2015	Shandong	Human	t899	XII
AH018	GCF_003310885.1	2015	Shandong	Human	t899	XII
AG037	GCF_003310905.1	2015	Shandong	Human	t899	XII
AF012	GCF_003310925.1	2015	Shandong	Human	Unknow	XII
1AK045	GCF_003311395.1	2015	Shandong	Human	t899	MSSA
S1AI048	GCF_003309535.1	2016	Shandong	Human	t899	MSSA
S1AH063	GCF_003309585.1	2016	Shandong	Human	t899	MSSA
S1AH053	GCF_003309595.1	2016	Shandong	Human	t899	MSSA
S1AH011	GCF_003309965.1	2016	Shandong	Human	t899	MSSA

### Comparative genome analysis

The annotation of the strains was carried out using the Prokka (rapid prokaryotic genome annotation). Snippy (Rapid haploid variant calling and core genome alignment) was used to compare the genome differences between ZY462471 and S1AH063 strains. The difference is displayed graphically using R language. The circular image and circular comparisons between multiple genomes and plasmids were performed by BLAST Ring Image Generator (BRIG)2. The TSAR01, NT-X55, and cp028191 strains were used as reference.

### Toxome and resistome profiles

Virulence genes were identified by blasting the VFDB database (http://www.mgc.ac.cn/VFs/main.htm), and the resistance genes was detected using the Comprehensive Antibiotic Resistance Database (CARD) (https://card.mcmaster.ca/).

### Survival rates of Galleria mellonella larvae infected with ZY462471

*S. aureus* strains were cultured in TSB with shaking at 220 rpm for 12 h at 37°C. Then strains were resuspended in PBS at an optical density at 600 nm of 0.8. After weighing, large *G. mellonella* larvae were paired and grouped so that each strain (ZY462471, S1AH063, ST239, ST5, USA300 and PBS [control], and blank) was of equal quality, with an average of about 300 mg larvae per strain. Then *G. mellonella larvae* were injected in the buttock with 5×10^4^ bacterial cells suspended in 50 μL NaCl solution. Then the treated larvae were placed in a clean, sterile petri dish at 25°C. We recorded the number of dead larvae for 40 h and calculate the survival rate.

### Analysis of haemolytic capacity

Haemolytic capacity was assessed as described previously [22]. Bacteria cultures were cultivated in TSB at 37°C with shaker. After 6 h of incubation, strains were centrifuged at 140000 ×*g* for 8 min to collect the suspension. Washed rabbit red blood cells (RRBCs) diluted at a concentration of 1% in 0.9% NaCl solution (pH 7.0) containing 0.1% bovine serum albumin (Sigma-Aldrich, St. Louis, Missouri, USA) was added to the new centrifuge tubes. Then, 20-μL supernatants were added to each tube and incubated at 37°C for 3 h. HA-MRSA ST239, HA-MRSA ST5, MSSA ST9 (S1AH063), and CA-MRSA strain USA300_FPR3757 were used in the haemolysis study for comparison.

### Analysis of cell viability by the lactate dehydrogenase assay

Human neutrophils were obtained from venous blood of healthy male adults per protocols [23] based on the regulations passed by the ethics committee of the First Affiliated Hospital of Zhejiang University, School of Medicine. All healthy volunteers provided informed consent before donating blood. Strains were grown for 6 h and the culture was diluted 1:100 and neutrophils were added in the 96-well microplate incubated at 37°C for 3 h. We used 0.1% Triton-X100 as the complete cytolysis group to determine 100% lysis. Cell viability was performed by using Cytotoxicity Detection Kit PLUS (LDH) (Roche, UK) in accordance with the manufacturer's protocol. To determine the percentage of cytotoxicity, it is necessary to calculate the average absorbance value of the triplicate sample and the control; the value was calculated as follows: Cytotoxicity (%)=$\frac{\left(\text{exp}.\;\mathrm{value}\;-\;\mathrm{low}\;\mathrm{control}\right)}{\left(\text{high control }-\;\mathrm{low}\;\mathrm{control}\right)}$×100. HA-MRSA ST239, HA-MRSA ST5, ST9 (S1AH063), and CA-MRSA strain USA300_FPR3757 were used for comparison.

### Mouse model of skin infection assay

Animal assays were approved by the Institutional Animal Care and Ethics Committee at The First Affiliated Hospital of Zhejiang University, College of Medicine. Six-week-old, female, BALB/C-nu mice were selected for this experiment. The mice were housed for a week prior to infection. Strains were grown for 9 h (the post-exponential phase), and washed thrice in normal saline solution. Then, mice were injected in the buttock with 1×10^8^ bacterial cells suspended in 100 μL NS solution. We checked lesions or abscesses on the skin of each mouse daily with callipers. The abscess size was calculated using length (L)×width (W). Skin abscess was measured and recorded for 6 days, after which all mice were implemented with euthanasia. Sterile NaCl solution was used as the negative control. MSSA ST9 S1AH063, HA-MRSA ST239, HA-MRSA ST5, and CA-MRSA strain USA300_FPR3757 and its *agr* mutant USA300△*agr* were used for comparison.

### Bacteriemia model caused by ZY462471

We used six-week-old, female, BALB/C mice to establish the bacteriemia model. Each mouse was housed for 10 days prior to injection. Strains were grown for 9 h were washed three times by NaCl solution, then 2×10^8^ bacterial cells were suspended in 100 μL sterile NaCl solution. Each mouse was injected with *S. aureus* cultures into the caudal vein (each group injected thirteen mice respectively). The negative control of each mouse was injected with 100 μL NaCl solution only. After inoculation, we monitored the physical condition of each mouse, and recorded every day. Once the first mouse infected by *S.aureus* strains died, we euthanized and dissected three mice with each group. Then we took the left kidney and homogenized in 1 ml of PBS. Then 200 μL of the homogenized tissue was diluted and plated on TSB agar to calculated the total counts of *S.aureus*. After 7 days of injection, the surviving mice was euthanized.

## Results and discussion

### Case report

The patient was a 65-year-old man, retired, in good health, and had a history of leprosy. He was admitted to the hospital with a 3-day history of fatigue. Physical examination revealed that his body temperature (T) was 38.5°C, the blood pressure (BP) was 134/71 mmHg, heart rate was 92 beats/min and breathing rate was 19 breaths/min. His right lower limb had a wound with fester and showed SSTI. Laboratory tests showed elevated C-reactive protein (CRP) level (195.8 mg/dL; normal range: <0.5 mg/dL) and a slightly elevated leukocyte count (10.89×10^9^/L; normal range: 4-10×10^9^/L), with a neutrophil ratio of 77.9%. The isolate was obtained from the patient’s blood, and identified as an ST9-MRSA strain. After the patient was admitted to the hospital, the abscess was incised and drained. He was treated a 1 g qd ivgtt with cefminox sodium. After the drug sensitivity result was reported, the patient was treated with vancomycin (one dose of 1000 mg) q12 h, and recovered and discharged after 2 weeks.

### Characteristics of ZY462471

We identified ZY462471 as a PVL-negative, t899-carrying, ST9 SCC*mec* XII isolate. Based on the definition of Centers for Disease Control and Prevention [1], this isolate was classified as a CA-MRSA strain. ST9-t899-MRSA XII is a predominant LA-MRSA clone in Asian countries, especially in the animal husbandry. A previous study from Taiwan reported that most ST9 isolates were shown to be resistant to ciprofloxacin, clindamycin, erythromycin, gentamicin, and tigecycline, suggesting that this multi-resistance was associated with the abuse of veterinary antimicrobial agents in the livestock industry in Asia [15]. In this study, ZY462471 was resistant to erythromycin, clindamycin, oxacillin, tetracycline, ciprofloxacin, levofloxacin, and gentamicin, but susceptible to vancomycin, rifampicin, moxifloxacin, trimethoprim-sulfamethoxazole, teicoplanin, amikacin, daptomycin, tigecycline, and linezolid. The minimum inhibitory concentrations (MICs) of erythromycin, tetracycline, gentamicin, and clindamycin assessed by broth microdilution method were 32 mg/L. Notably, the MIC of oxacillin was 4 mg/L, which is the cut-off value used for MRSA classification (Table 2).

**Table 2.** Antimicrobial susceptibility testing of ZY462471.

Antimicrobial agent	MIC (mg/l)	Result
Erythromycin	32	R
Clindamycin	32	R
Oxacillin	4	R
Trimethoprim/sulfamethoxazole	0.25	S
Tetracycline	32	R
Vancomycin	0.5	S
Rifampin	0.07	S
Ciprofloxacin	16	R
Levofloxacin	4	S
Moxifloxacin	0.5	S
Gentamicin	32	R
Teicoplanin	0.25	S
Amikacin	8	S
Tigecycline	0.5	S
Daptomycin	0.5	S
Linezolid	2	S

MIC, minimum inhibitory concentration; R, resistant; S, susceptible.

### Probable host evolutionary origins of ZY462471

**Figure 1.**
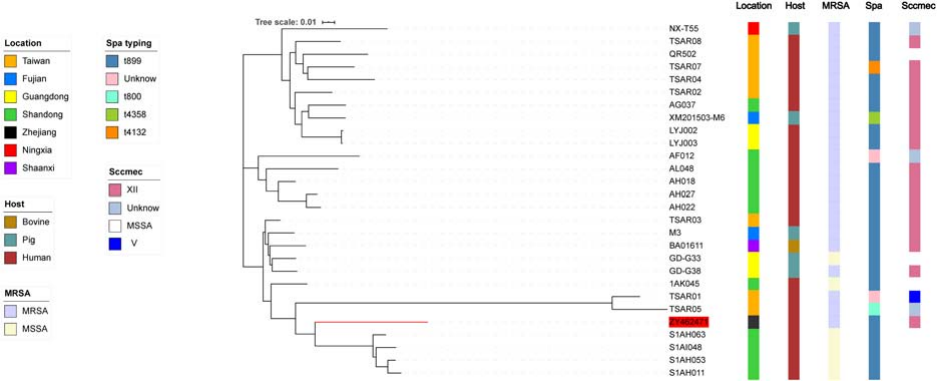
Construction of phylogenetic tree among ST9 isolates. Twenty-eight ST9 strains genome sequences were aligned. The information of ST9 strains is shown on the left, including the location, host, and the presence of SCC*mec* type and spa type. To reduce the number of parameters in the model, we grouped samples into seven geographical regions: Taiwan, Shandong, Fujian, Zhejiang, Shaanxi, Ningxia, and Guangdong. To explore putative host switching in ST9, we also added the sampled host as a discrete trait to the model. Three possible hosts were included human, bovine, and pig.

To investigate the evolutionary history of ZY462471 and identify potential ancestral host and geographical reservoirs, MEGAX was used to perform the phylogenetic reconstruction of the ST9 lineage in China, within the framework of the larger dataset. Based on the core gene sequence, we constructed a phylogenetic tree with our ST9 isolate ZY462471 and the other 27 reference isolates. As shown in Figure 1, ST9 in China includes three different clades, which is a human-adapted clade, a livestock-adapted clade, and human-livestock mix clade, dating back to 1998 and 2017. Most of them were t899, SCC*mec* XII.

As shown in Figure 1, our isolate was found in the human-livestock mix clade, which includes two different subclades. Our isolate was in the subcluster of six ST9 isolates from human-associated (TSAR01, TRSA05, S1AH063, S1AI048, S1AH053, and S1AH011) samples. Phylogenetic analysis showed that ZY467421 was more closely related to the S1AH063, S1AI048, S1AH053, and S1AH011 ST9 isolates from Shandong than the TSAR01 and TSAR05 isolates from Taiwan. However, S1AH063, S1AI048, S1AH053, and S1AH011 all belonged to MSSA strains, while ZY467421, TSAR01, and TSAR05 were all MRSA strains. Genetic study of LA-MRSA CC398 revealed that LA-MRSA CC398 originated from human-adapted MSSA clone, subsequently spread to livestock, and showed uptake of the SCC*mec* cassette and resultant methicillin resistance in some cases [16]. Moreover, He et al. [24] reported several cases of severe community infections with CA-MRSA ST398 and proposed that the CA-MRSA ST398 isolates evolved from human-adapted MSSA strains rather than from a livestock-adapted isolate. Hence, based on our result of phylogenetic analysis we speculated that the ZY467421 strain may have evolved from human-adapted, methicillin-susceptible ST9 clones that spread to livestock, and then acquired resistance to ciprofloxacin, clindamycin, erythromycin, and gentamicin in the livestock industry. Subsequently, the strain acquired methicillin resistance on some occasions.

### Comparison of the genomic in ZY462471 and S1AH063

**Figure 2.**
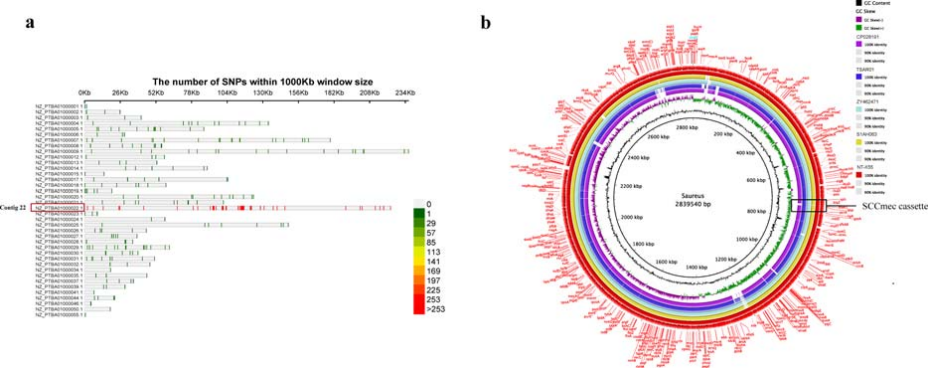
Comparison of the chromosome of ZY46241 and S1AH063. (**a**) shows the distribution of the different SNPs between our strain ZY462471 and S1AH063 on different contigs. There are totally thirty-eight contigs. The colour in the lower right corner represents the number of nucleic acid variation on different contigs. (**b**) shows the chromosomal difference between ZY462471 and S1AH063. Each circle represents the chromosomes of different strains, and the blank places indicate differences. TSAR01, NT-X55, and cp028191 were used as reference strains. Strains are grouped in different colours. The SCC*mec* cassette is marked in black frames.

Based on the results of phylogenetic reconstruction in this study, S1AH063 showed the closest relationship to ZY462471. Therefore, we chose S1AH063 for further investigation. Compared to S1AH063, we found 479 nucleotide variations in ZY462471 and S1AH063, including 385 SNP, 67 com, and 18 del. As shown in Figure 2(a) most of these mutations are concentrated in contig22 (255 pieces), which has a fragment length of about 240 K and a total of 239 CDS. The function of these CDS is mainly related to DNA function, including DNA replication, DNA repair, uncoiling, DNA recombination and extension, transposon insertion, and endonuclease and heat-inducible transcription repressor (Table S1). This indicated that the ZY462471 strain can mutate more easily than S1AH063 in these places, thereby gaining or losing some genes. As shown in Figure 2(b), the backbone of ZY462471 is almost identical to that of S1AH063 rather than of other reference strains. The white area represents the missing part. Of note, in the SCC*mec* cassette region, we observed an obvious deletion of the SCC*mec* cassette. It is speculated that our strain obtained SCC*mec* from this MSSA strain and thus gained methicillin resistance.

### Analysis and comparison of antimicrobial resistome and toxome profiles

**Figure 3.**
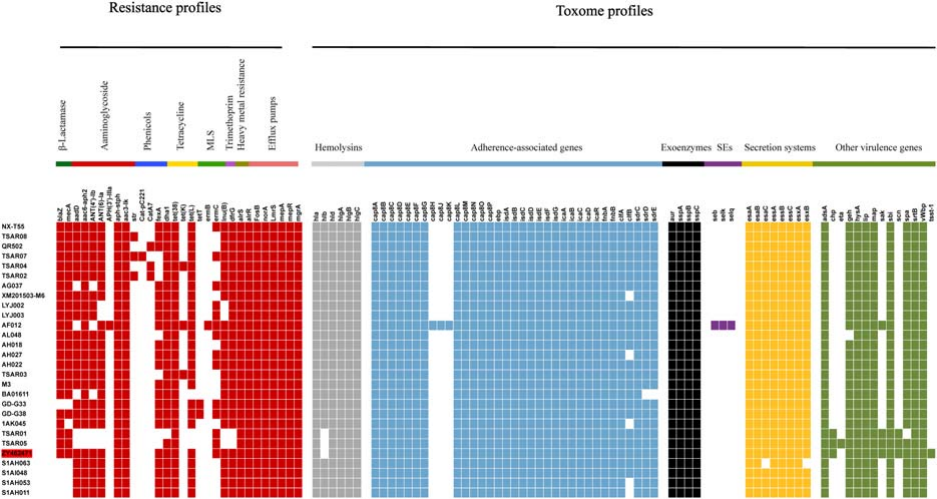
The heatmap of antibiotic resistance gene proﬁles and virulence genes across the 28 ST9 *S. aureus* isolates. Coloured blocks represent the presence of genes and white blocks represent absence. The horizontal colour bar from left to right represents the involved genes in β-lactamase, aminoglycoside, phenicols, tetracycline, MLS, trimethoprim, heavy metal resistance, efflux pumps, haemolysins, adherence-associated genes, exoenzymes, *staphylococcal* enterotoxins, secretion systems, and other virulence genes. The strain ZY462471 used in this study was marked with red colour. The detailed information of other 27 ST isolates was shown in Table 1.

The different distributions of the virulence factors and resistance genes carried in the ST9 isolates are shown in Figure 3. All ST9 isolates showed multidrug resistance pattern and harboured multiple drug-resistant genes. Consistent with the results of the phylogenetic analysis, the ZY462471 and S1AH063, S1AI048, S1AH053, and S1AH011 harboured the similar resistance profiles except harboured the *mecA*, *blaZ* and *ermC* genes.

In the toxome profiles, ZY62471 was found harbouring the chemotaxis inhibitory protein (*chp*), staphylococcal complement inhibitor (*scn*), and staphylokinase (*sak*) human evasion genes, which are not typically found in LA-MRSA strains. The *S. aureus* IEC harboures the *scn*, *sak*, *sea,* and *chp* genes, which is encoded by β-haemolysin-converting bacteriophages (βC-Φs) [25]. Seven IEC variants have been identified [26]. A previous study showed that *scn*-positive *S. aureus* is unique in humans, in that it plays an important role in inhibiting phagocytosis by neutrophils [27]. Rooijakkers et al. [28] also reported that *scn* cannot affect the complement formation in animals such as sheep, pig, cow, and goat. *chp* encodes a modulator of chemotactic factors, which can suppress both chemotaxis and activation by neutrophils [29]. *sak* encodes an anti-opsonin and inhibitor of defensins [30]. All these above-mentioned IEC factors showed a specific effect on the human immune system rather than on the animal responses [28]. Recent evidence of the origin of CC398 has shown that the absence of IEC genes on βC-Φs was likely related to animal specificity, while IEC-positive clones may be associated with human specificity [16,31,32]. However, this human-specificity relation in ST9 is still uncertain. Dan et al. [33] investigated 139 ST9 strains isolated from 1458 pigs and found that all ST9 isolates from pigs lacked IEC genes, indicating that missing IEC genes may be associated with pig specificity. In this study, except the ZY462471 isolate and other two strains (TSAR01 and TSAR05), all ST9 isolates showed absence of *sak*, *scn,* and *chp* genes, suggesting that the IEC genes may be the genetic determinants associated with human origin and adaptation. Moreover, bacteriophages are mobile genetic elements and result in the horizontal transfer of the IEC genes between bacteria. Therefore, we have more reason to speculate that ZY4762471 originated from human clinical *S. aureus* strains containing an IEC-carrying βC-Φ on some occasions.

Of note, the bacteriophages carrying IEC genes are integrated into the *hlb* gene which can encode β-haemolysin. Thus, the IEC-positive *S. aureus* clones often lacked the *hlb* gene, thereby indicating that β-haemolysin could not be produced. In this study, the ST9 MRSA isolate ZY62471 did not harbour the *hlb* gene.

These results were consistent with the fact that this patient was infected with ST9-t899-XII, IEC-positive CA-MRSA strain without animal contact. However, we cannot exclude the possibility that this ST9 isolate may have been acquired from other sources.

### Virulence assays analysis

Previous studies have reported that *mecA* shows repression of virulence [34,35]; these investigations prompted us to evaluate a possible role of the SCC*mec* cassette of CA-MRSA virulence. Hence, the virulence potential between MSSA ST9 strain (S1AH063) and MRSA ST9 ZY462471 was investigated. Furthermore, the predominant Chinese HA-MRSA strains (ST5, ST239) and the CA-MRSA clone USA300_FPR3757 were also used for comparison.

**Figure 4.**
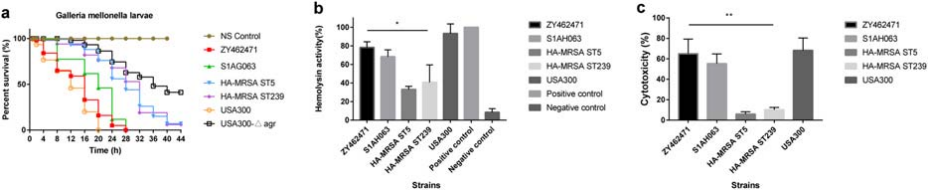
Virulence phenotype of MRSA ST9 isolate ZY462471. (**a**) The Survival rates of ZY42671 infected with *Galleria mellonella* larvae. One hundred larvae were used per strain. *G. mellonella* larvae were inoculated with 50 µl each strain at doses ranging from 5×10^4^ and incubated at 37°C; the viability was assessed over 40 h. (**b**) The Triton X-100 solution group (added to 1% rabbit RBCs) was used as the positive control, and 0.9% NaCl solution was used as the negative control. The absorbance at 600 nm of each sample was recorded. All data were calibrated with negative controls. (**c**) The cytotoxicity assay was performed in accordance with the following controls. (1) exp. value: the absorbance value of each experimental sample. (2) low control: the absorbance value of culture medium background. (3) high control: the absorbance value of the positive control (maximum LDH release). Values are indicated as means ± SD (three repeated experiments). * represents *p* < 0.05. ** represents *p* < 0.01.

To evaluate the virulence potential of ZY462471, we first compared survival rates of *G. mellonella* infected with ZY462471, S1AH063, the high-virulent strain USA300 and low-virulent strains HA-MRSA ST239 and ST5 to evaluate differences in pathogenicity. As shown in Figure 4(a), the percentage of surviving larvae was much lower following infection with ZY462571, S1AH063 and USA300 as compared to HA-MRSA strains and USA300△*agr* mutant (p<0.01). The ZY462471 strain was more virulent than the HA-MRSA ST239 strain and HA-MRSA ST5 strain (*G. mellonella* survival ≤60% at 12 h and ≤3% at 28 h).

Haemolysis is one of the most important determinants of virulence in *S. aureus*. Accordingly, we first compared the haemolysin ability of these strains. As shown in Figure 4(b), the ST9 CA-MRSA isolate ZY462471 had significantly stronger haemolytic ability than HA-MRSA, suggesting that ZY462471 has much higher virulence level than HA-MRSA clones. The haemolytic abilities of ZY462471 and the human ST9 MSSA clone were comparable, indicating that the evolution of ST9 MRSA is not accompanied by a decline in virulence.

Human neutrophils are a part of the immune system, which form the first line of defense against *S. aureus* infections [36]. Therefore, to determine the virulence potential of ZY462471, we evaluated the capacity of ZY462471, HA-MRSA strains (ST5 and ST239), and the CA-MRSA USA300_FPR3757 to lyse human neutrophils after phagocytosis. As shown in Figure 4(c), the degree of neutrophil lysis caused by ZY462471 was much higher than that by HA-MRSA strains (P<0.05), and slightly lower than that of the CA-MRSA USA300 clone. However, ZY462471 is slightly more cytotoxic than the ST9 MSSA strain (S1AH063). In summary, ZY462471 was shown to have higher cytolytic capacity than HA-MRSA strains, but was similar to those of the USA300 and MSSA ST9 strains.

### The mouse skin infection model and the mouse blood stream infection model caused by ZY462417

**Figure 5.**
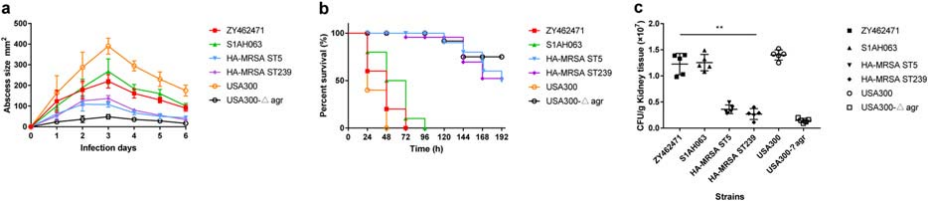
The mouse systemic infection model and skin infection model caused by ZY462471 compared with that of caused by its closest ST9 MSSA neighbour S1AH063 and HA-MRSA ST5 strain, HA-MRSA ST239 strain, and CA-MRSA USA300 and its *agr* mutant USA300△*agr*. (**a**) Abscess sizes in the mouse skin infection model. Three mice were used per strain. (**b**) Survival analysis of mice (*n* = 10 per strain) injected with 2×10^8^ CFU of strains or NaCl solution. Survival curves were compared using a log-rank (Mantel-Cox) test. (**c**) The number of CFUs in the kidneys was determined by plating samples on TSB agar.

Based on case reports, the primary disease caused by ZY462471 was skin and soft tissue infection. Therefore, mouse skin infection model was used to evaluate the invasive capacity of ZY462741 *in vivo*. USA300△*agr* mutant strain was used as a negative control. A low-virulence HA-MRSA ST239 and ST5 strains were used for comparison. As shown in Figure 5(a), the size of skin abscesses in mice infected by ZT462471 was significantly larger than those infected by HA-MRSA (ST239 and ST5) and the USA300△*agr* mutant (p<0.05), but smaller than those caused by the S1AH063 isolate (p>0.05). This finding revealed that the ZY462471 strain has strong potential to cause invasive skin infections, suggesting that ZY62471 was highly virulent. It is important to emphasize that CA-MRSA clones are not always more virulent than many MSSA strains. A previous study from North America proposed that the virulence of NCTC8325 clone (an ST8 MSSA strain) was similar to that of CA-MRSA strains such as USA300 [37]. Further, SSTIs are the most frequent condition associated with CA-MRSA, which lead to at least 90% of CA-MRSA infections [38]. In this study, the patient had SSTI in his leg at first, followed by bacterial invasion of the bloodstream, leading to septicaemia.

After evaluating the invasive capacity of ZY462471, we then used bacteremia models to explore its pathogenicity. As shown in Figure 5(b), in the bacteremia model, the mice infected with USA300, ZY462471 and S1AH063 were all died with 96 h (n=10). Infection with the ZY462471 produced significantly greater mortality compared with the HA-MRSA strain ST239, ST5 strains and USA300△*agr* mutant. As shown in Figure 5(c), the mice infected with ZY462471 showed much higher CFU counts in the kidneys than the mice infected with the ST239, ST5 strains and the USA300△*agr* mutant. These findings demonstrate the high virulence of CA-MRSA isolate ZY4624171.

The results of all these virulence assays showed that the ST9 CA-MRSA isolate ZY462471 had similar high virulence as the likely ancestral MSSA ST9 clone S1AH063. However, previous studies have suggested that the attenuation of virulence is a trade-off of the strain under the stress of antibiotic treatment, and the acquisition of drug resistance genes or elements is inversely correlated with the virulence levels. In this study, the CA-MRSA ST9 ZY462471 could only grow at <4 μg/mL oxacillin, suggesting that the ST9 CA-MRSA ZY462471 with a very low methicillin resistance level had most likely evolved from MSSA strains and notably, without the attenuation of virulence.

## Conclusion

We identified one ST9 CA-MRSA strain causing bloodstream infection. Unlike LA-MRSA ST9, this CA-MRSA ST9 isolate—ZY462471—showed high virulence, and phylogenetic analysis showed that ZY462541 probably evolved from human-adapted MSSA ST9 strains by acquiring the SCC*mec* cassette with methicillin resistance, and further achieved resistance to ciprofloxacin, clindamycin, erythromycin, and gentamicin through the livestock industry. The clinical information and genomic results suggested that no clonal transmission occurred in this case. This patient denied livestock contact, and further investigations of virulence factors showed that the isolate had characteristics of human-associated isolates (containing IEC genes). A major limitation of the study is that we detected only one ST9 CA-MRSA strain. This means that the transmission tendency and the potential threat of this clone is difficult to evaluate, both in the community and in medical institutions. Further investigations should be performed to clarify how the new ST9 isolate evolved to combine high virulence with methicillin resistance. Further, the mechanism of action of gaining βC-Φs from human clinical *S. aureus* strains also warrants further investigation.

## Supplementary Material

Table_S1.docx
