# Supplementary material for: Characterization of highly virulent community-associated methicillin-resistant Staphylococcus aureus ST9-SCCmec XII causing bloodstream infection in China
Source: Emerg Microbes Infect. 2020 Dec 1;9(1):2526–35. doi: 10.1080/22221751.2020.1848354 (PMC7717876; doi:10.1080/22221751.2020.1848354)
Supplement: Table_S1.docx [file TEMI_A_1848354_SM4789.docx]

**Table S1. Non-synonymous SNPs in contig22 in ZY462471 compared with the closest MSSA ST9 isolate.**

| **locus_tag** | **Gene** | **Product** |
| --- | --- | --- |
| GGHEMGKM_00001 | rnhA | 14.7 kDa ribonuclease H-like protein |
| GGHEMGKM_00002 | ebh | Extracellular matrix-binding protein ebh |
| GGHEMGKM_00003 | norB | Quinolone resistance protein NorB |
| GGHEMGKM_00004 | steT | Serine/threonine exchanger SteT |
| GGHEMGKM_00005 | tdcB | L-threonine dehydratase catabolic TdcB |
| GGHEMGKM_00006 | ald1 | Alanine dehydrogenase 1 |
| GGHEMGKM_00007 | ypcP | 5'-3' exonuclease |
| GGHEMGKM_00008 | der_1 | GTPase Der |
| GGHEMGKM_00009 | | hypothetical protein |
| GGHEMGKM_00010 | | hypothetical protein |
| GGHEMGKM_00011 | rlmL | Ribosomal RNA large subunit methyltransferase L |
| GGHEMGKM_00012 | | hypothetical protein |
| GGHEMGKM_00013 | gpsB | Cell cycle protein GpsB |
| GGHEMGKM_00014 | | hypothetical protein |
| GGHEMGKM_00015 | | hypothetical protein |
| GGHEMGKM_00016 | recU | Holliday junction resolvase RecU |
| GGHEMGKM_00017 | ponA | Penicillin-binding protein 1A/1B |
| GGHEMGKM_00018 | | hypothetical protein |
| GGHEMGKM_00019 | nth | Endonuclease III |
| GGHEMGKM_00020 | dnaD | DNA replication protein DnaD |
| GGHEMGKM_00021 | asnS | Asparagine--tRNA ligase |
| GGHEMGKM_00022 | dinG | putative ATP-dependent helicase DinG |
| GGHEMGKM_00023 | birA | Bifunctional ligase/repressor BirA |
| GGHEMGKM_00024 | cca | CCA-adding enzyme |
| GGHEMGKM_00025 | bshA | N-acetyl-alpha-D-glucosaminyl L-malate synthase |
| GGHEMGKM_00026 | | hypothetical protein |
| GGHEMGKM_00027 | | hypothetical protein |
| GGHEMGKM_00028 | | hypothetical protein |
| GGHEMGKM_00029 | | hypothetical protein |
| GGHEMGKM_00030 | lapB | Lipopolysaccharide assembly protein B |
| GGHEMGKM_00031 | aroA | 3-phosphoshikimate 1-carboxyvinyltransferase |
| GGHEMGKM_00032 | aroB | 3-dehydroquinate synthase |
| GGHEMGKM_00033 | aroC | Chorismate synthase |
| GGHEMGKM_00034 | | hypothetical protein |
| GGHEMGKM_00035 | ndk | Nucleoside diphosphate kinase |
| GGHEMGKM_00036 | hepT | Heptaprenyl diphosphate synthase component 2 |
| GGHEMGKM_00037 | menG | Demethylmenaquinone methyltransferase |
| GGHEMGKM_00038 | | hypothetical protein |
| GGHEMGKM_00039 | hup | DNA-binding protein HU |
| GGHEMGKM_00040 | gpsA | Glycerol-3-phosphate dehydrogenase [NAD(P)+] |
| GGHEMGKM_00041 | der_2 | GTPase Der |
| GGHEMGKM_00042 | rpsA | 30S ribosomal protein S1 |
| GGHEMGKM_00043 | cmk | Cytidylate kinase |
| GGHEMGKM_00044 | ansA | putative L-asparaginase |
| GGHEMGKM_00045 | | Glucosaminate ammonia-lyase |
| GGHEMGKM_00046 | ebpS | Elastin-binding protein EbpS |
| GGHEMGKM_00047 | recQ | ATP-dependent DNA helicase RecQ |
| GGHEMGKM_00048 | | hypothetical protein |
| GGHEMGKM_00049 | | Ferredoxin |
| GGHEMGKM_00050 | ribU | Riboflavin transporter RibU |
| GGHEMGKM_00051 | | hypothetical protein |
| GGHEMGKM_00052 | | hypothetical protein |
| GGHEMGKM_00053 | | hypothetical protein |
| GGHEMGKM_00054 | | hypothetical protein |
| GGHEMGKM_00055 | | hypothetical protein |
| GGHEMGKM_00056 | lytN | putative cell wall hydrolase LytN |
| GGHEMGKM_00057 | | hypothetical protein |
| GGHEMGKM_00058 | | hypothetical protein |
| GGHEMGKM_00059 | | hypothetical protein |
| GGHEMGKM_00060 | | hypothetical protein |
| GGHEMGKM_00061 | | hypothetical protein |
| GGHEMGKM_00062 | | hypothetical protein |
| GGHEMGKM_00063 | | hypothetical protein |
| GGHEMGKM_00064 | | hypothetical protein |
| GGHEMGKM_00065 | | hypothetical protein |
| GGHEMGKM_00066 | | Glycyl-glycine endopeptidase ALE-1 |
| GGHEMGKM_00067 | | hypothetical protein |
| GGHEMGKM_00068 | | hypothetical protein |
| GGHEMGKM_00069 | | hypothetical protein |
| GGHEMGKM_00070 | | hypothetical protein |
| GGHEMGKM_00071 | | hypothetical protein |
| GGHEMGKM_00072 | | hypothetical protein |
| GGHEMGKM_00073 | | hypothetical protein |
| GGHEMGKM_00074 | | hypothetical protein |
| GGHEMGKM_00075 | | hypothetical protein |
| GGHEMGKM_00076 | clpP1 | ATP-dependent Clp protease proteolytic subunit 1 |
| GGHEMGKM_00077 | | hypothetical protein |
| GGHEMGKM_00078 | | hypothetical protein |
| GGHEMGKM_00079 | | hypothetical protein |
| GGHEMGKM_00080 | | hypothetical protein |
| GGHEMGKM_00081 | | hypothetical protein |
| GGHEMGKM_00082 | | hypothetical protein |
| GGHEMGKM_00083 | | hypothetical protein |
| GGHEMGKM_00084 | | hypothetical protein |
| GGHEMGKM_00085 | | hypothetical protein |
| GGHEMGKM_00086 | | hypothetical protein |
| GGHEMGKM_00087 | | hypothetical protein |
| GGHEMGKM_00088 | | hypothetical protein |
| GGHEMGKM_00089 | | hypothetical protein |
| GGHEMGKM_00090 | | hypothetical protein |
| GGHEMGKM_00091 | | hypothetical protein |
| GGHEMGKM_00092 | | hypothetical protein |
| GGHEMGKM_00093 | | hypothetical protein |
| GGHEMGKM_00094 | | hypothetical protein |
| GGHEMGKM_00095 | | hypothetical protein |
| GGHEMGKM_00096 | | hypothetical protein |
| GGHEMGKM_00097 | | hypothetical protein |
| GGHEMGKM_00098 | | hypothetical protein |
| GGHEMGKM_00099 | | hypothetical protein |
| GGHEMGKM_00100 | | hypothetical protein |
| GGHEMGKM_00101 | | hypothetical protein |
| GGHEMGKM_00102 | | hypothetical protein |
| GGHEMGKM_00103 | | hypothetical protein |
| GGHEMGKM_00104 | dnaC | DNA replication protein DnaC |
| GGHEMGKM_00105 | | hypothetical protein |
| GGHEMGKM_00106 | | hypothetical protein |
| GGHEMGKM_00107 | ssbA | Single-stranded DNA-binding protein A |
| GGHEMGKM_00108 | | hypothetical protein |
| GGHEMGKM_00109 | | hypothetical protein |
| GGHEMGKM_00110 | | hypothetical protein |
| GGHEMGKM_00111 | | hypothetical protein |
| GGHEMGKM_00112 | | hypothetical protein |
| GGHEMGKM_00113 | | hypothetical protein |
| GGHEMGKM_00114 | | hypothetical protein |
| GGHEMGKM_00115 | | hypothetical protein |
| GGHEMGKM_00116 | | hypothetical protein |
| GGHEMGKM_00117 | | hypothetical protein |
| GGHEMGKM_00118 | | hypothetical protein |
| GGHEMGKM_00119 | | hypothetical protein |
| GGHEMGKM_00120 | | hypothetical protein |
| GGHEMGKM_00121 | | hypothetical protein |
| GGHEMGKM_00122 | | hypothetical protein |
| GGHEMGKM_00123 | | hypothetical protein |
| GGHEMGKM_00124 | | hypothetical protein |
| GGHEMGKM_00125 | Int-Tn | Transposase from transposon Tn916 |
| GGHEMGKM_00126 | | hypothetical protein |
| GGHEMGKM_00127 | | hypothetical protein |
| GGHEMGKM_00128 | srrB | Sensor protein SrrB |
| GGHEMGKM_00129 | srrA | Transcriptional regulatory protein SrrA |
| GGHEMGKM_00130 | rluB | Ribosomal large subunit pseudouridine synthase B |
| GGHEMGKM_00131 | scpB | Segregation and condensation protein B |
| GGHEMGKM_00132 | scpA | Segregation and condensation protein A |
| GGHEMGKM_00133 | | hypothetical protein |
| GGHEMGKM_00134 | xerD | Tyrosine recombinase XerD |
| GGHEMGKM_00135 | fur | Ferric uptake regulation protein |
| GGHEMGKM_00136 | nudF | ADP-ribose pyrophosphatase |
| GGHEMGKM_00137 | yhdN | General stress protein 69 |
| GGHEMGKM_00138 | | hypothetical protein |
| GGHEMGKM_00139 | | putative oxidoreductase |
| GGHEMGKM_00140 | proC | Pyrroline-5-carboxylate reductase |
| GGHEMGKM_00141 | rnz | Ribonuclease Z |
| GGHEMGKM_00142 | zwf | Glucose-6-phosphate 1-dehydrogenase |
| GGHEMGKM_00143 | marA | Multiple antibiotic resistance protein MarA |
| GGHEMGKM_00144 | malL | Oligo-1,6-glucosidase |
| GGHEMGKM_00145 | malR | HTH-type transcriptional regulator MalR |
| GGHEMGKM_00146 | | hypothetical protein |
| GGHEMGKM_00147 | | putative multidrug-efflux transporter |
| GGHEMGKM_00148 | gnd | 6-phosphogluconate dehydrogenase, decarboxylating |
| GGHEMGKM_00149 | pepT | Peptidase T |
| GGHEMGKM_00150 | | hypothetical protein |
| GGHEMGKM_00151 | | hypothetical protein |
| GGHEMGKM_00152 | pdhC | Dihydrolipoyllysine-residue acetyltransferase component of pyruvate dehydrogenase complex |
| GGHEMGKM_00153 | bfmBAB | 2-oxoisovalerate dehydrogenase subunit beta |
| GGHEMGKM_00154 | bfmBAA | 2-oxoisovalerate dehydrogenase subunit alpha |
| GGHEMGKM_00155 | | Dihydrolipoyl dehydrogenase |
| GGHEMGKM_00156 | recN | DNA repair protein RecN |
| GGHEMGKM_00157 | argR | Arginine repressor |
| GGHEMGKM_00158 | | Farnesyl diphosphate synthase |
| GGHEMGKM_00159 | xseB | Exodeoxyribonuclease 7 small subunit |
| GGHEMGKM_00160 | xseA | Exodeoxyribonuclease 7 large subunit |
| GGHEMGKM_00161 | nusB | N utilization substance protein B |
| GGHEMGKM_00162 | | hypothetical protein |
| GGHEMGKM_00163 | cfiB | 2-oxoglutarate carboxylase small subunit |
| GGHEMGKM_00164 | accB | Biotin carboxyl carrier protein of acetyl-CoA carboxylase |
| GGHEMGKM_00165 | efp | Elongation factor P |
| GGHEMGKM_00166 | ypdF | Aminopeptidase YpdF |
| GGHEMGKM_00167 | | hypothetical protein |
| GGHEMGKM_00168 | | hypothetical protein |
| GGHEMGKM_00169 | lipM | Octanoyltransferase LipM |
| GGHEMGKM_00170 | moeZ | putative adenylyltransferase/sulfurtransferase MoeZ |
| GGHEMGKM_00171 | gcvPB | putative glycine dehydrogenase (decarboxylating) subunit 2 |
| GGHEMGKM_00172 | gcvPA | putative glycine dehydrogenase (decarboxylating) subunit 1 |
| GGHEMGKM_00173 | gcvT | Aminomethyltransferase |
| GGHEMGKM_00174 | aroK | Shikimate kinase |
| GGHEMGKM_00175 | | hypothetical protein |
| GGHEMGKM_00176 | | hypothetical protein |
| GGHEMGKM_00177 | | hypothetical protein |
| GGHEMGKM_00178 | comGC | ComG operon protein 3 |
| GGHEMGKM_00179 | epsF | Type II secretion system protein F |
| GGHEMGKM_00180 | comGA | ComG operon protein 1 |
| GGHEMGKM_00181 | | putative metallo-hydrolase |
| GGHEMGKM_00182 | | hypothetical protein |
| GGHEMGKM_00183 | glcK | Glucokinase |
| GGHEMGKM_00184 | | hypothetical protein |
| GGHEMGKM_00185 | gluP | Rhomboid protease GluP |
| GGHEMGKM_00186 | | 5-formyltetrahydrofolate cyclo-ligase |
| GGHEMGKM_00187 | rpmG2 | 50S ribosomal protein L33 2 |
| GGHEMGKM_00188 | pbpH | Penicillin-binding protein H |
| GGHEMGKM_00189 | sodA | Superoxide dismutase [Mn] 1 |
| GGHEMGKM_00190 | zur | Zinc-specific metallo-regulatory protein |
| GGHEMGKM_00191 | znuB | High-affinity zinc uptake system membrane protein ZnuB |
| GGHEMGKM_00192 | znuC | High-affinity zinc uptake system ATP-binding protein ZnuC |
| GGHEMGKM_00193 | nfo | putative endonuclease 4 |
| GGHEMGKM_00194 | cshB | DEAD-box ATP-dependent RNA helicase CshB |
| GGHEMGKM_00195 | | GTP cyclohydrolase 1 type 2 |
| GGHEMGKM_00196 | trmK | tRNA (adenine(22)-N(1))-methyltransferase |
| GGHEMGKM_00197 | sigA | RNA polymerase sigma factor SigA |
| GGHEMGKM_00198 | dnaG | DNA primase |
| GGHEMGKM_00199 | yqfL | Putative pyruvate, phosphate dikinase regulatory protein |
| GGHEMGKM_00200 | ccpN | Transcriptional repressor CcpN |
| GGHEMGKM_00201 | glyQS | Glycine--tRNA ligase |
| GGHEMGKM_00202 | recO | DNA repair protein RecO |
| GGHEMGKM_00203 | era | GTPase Era |
| GGHEMGKM_00204 | cdd | Cytidine deaminase |
| GGHEMGKM_00205 | dgkA | Undecaprenol kinase |
| GGHEMGKM_00206 | ybeY | Endoribonuclease YbeY |
| GGHEMGKM_00207 | | PhoH-like protein |
| GGHEMGKM_00208 | | hypothetical protein |
| GGHEMGKM_00209 | | hypothetical protein |
| GGHEMGKM_00210 | | hypothetical protein |
| GGHEMGKM_00211 | rpsU | 30S ribosomal protein S21 |
| GGHEMGKM_00212 | mtaB | Threonylcarbamoyladenosine tRNA methylthiotransferase MtaB |
| GGHEMGKM_00213 | rsmE | Ribosomal RNA small subunit methyltransferase E |
| GGHEMGKM_00214 | prmA | Ribosomal protein L11 methyltransferase |
| GGHEMGKM_00215 | dnaJ | Chaperone protein DnaJ |
| GGHEMGKM_00216 | dnaK | Chaperone protein DnaK |
| GGHEMGKM_00217 | grpE | Protein GrpE |
| GGHEMGKM_00218 | hrcA | Heat-inducible transcription repressor HrcA |
| GGHEMGKM_00219 | hemN | Oxygen-independent coproporphyrinogen-III oxidase-like protein YqeR |
| GGHEMGKM_00220 | lepA | Elongation factor 4 |
| GGHEMGKM_00221 | rpsT | 30S ribosomal protein S20 |
| GGHEMGKM_00222 | | hypothetical protein |
| GGHEMGKM_00223 | comEC | ComE operon protein 3 |
| GGHEMGKM_00224 | tadA | tRNA-specific adenosine deaminase |
| GGHEMGKM_00225 | comEA | ComE operon protein 1 |
| GGHEMGKM_00226 | tylM1 | dTDP-3-amino-3,6-dideoxy-alpha-D-glucopyranose N,N-dimethyltransferase |
| GGHEMGKM_00227 | rsfS | Ribosomal silencing factor RsfS |
| GGHEMGKM_00228 | | hypothetical protein |
| GGHEMGKM_00229 | nadD | putative nicotinate-nucleotide adenylyltransferase |
| GGHEMGKM_00230 | yhbY | RNA-binding protein YhbY |
| GGHEMGKM_00231 | aroE | Shikimate dehydrogenase (NADP(+)) |
| GGHEMGKM_00232 | der_3 | GTPase Der |
| GGHEMGKM_00233 | gph | Phosphoglycolate phosphatase |
| GGHEMGKM_00234 | mtnN | 5'-methylthioadenosine/S-adenosylhomocysteine nucleosidase |
| GGHEMGKM_00235 | | hypothetical protein |
| GGHEMGKM_00236 | entA | Enterotoxin type A |
| GGHEMGKM_00237 | entD | Enterotoxin type D |
| GGHEMGKM_00238 | | hypothetical protein |
| GGHEMGKM_00239 | | hypothetical protein |
